# The protective effects of TanIIA on neurotoxicity induced by β-amyloid protein through the Cdk5/P35 pathway in cultured primary neurons

**DOI:** 10.1186/1750-1326-7-S1-S8

**Published:** 2012-02-07

**Authors:** Lili Shi, Weina Yang, Yihua Qian, Jianshui Zhang, Xinlin Chen, Yong Liu

**Affiliations:** 1 Department of Human Anatomy and Histology Embryology, Institute of Neurobiology, Key Laboratory of Environment and Genes Related to Diseases of Education Ministry, Xi’an Jiaotong University College of Medicine, China

## Background

The characteristic pathological changes of Alzheimer’s disease (AD) include extracellular deposits of β-amyloid protein (Aβ) in brain, intracellular neurofibrillary tangles (NFTs), as well as a few neuronal loss. Several lines of evidence show that, neurotoxicity, such as Aβ, glutamate, and oxidative stress, can lead to calcium influx, then induce the cleavage of P35 into P25. P25 is not readily degraded, and binding of p25 to Cyclin-dependent kinase 5 (Cdk5) constitutively activates Cdk5, changes its cellular location and alters its substrate specificity. The findings indicated that the p25/Cdk5 complex hyperphosphorylates tau which diminishes tau's ability to associate with microtubules, disrupts the cytoskeleton and promotes the apoptosis of neurons. The level of P25 have been found to be elevated in brain of AD and overexpression of P25 in a transgenic mouse results in the formation of phosphorylated tau, neurofibrillary tangles and cognitive deficits. Thus, reducing intracellular calcium ion concentration, inhibiting the cleavage of P35 into P25 may be a new target for AD. Tanshinone IIA(Tan IIA) is one of ingredients of Tanshinone, appears to have neuroprotective effects. Our previous study indicated that Tan IIA can reduce intracellular calcium ion concentration, and has antioxidant effects. Yet, we presumed that Tan IIA may have effects on neurodegenerative diseases, such as AD and so on.

## Methods

Primary cortical neuron culture combining 3-(4, 5-dimthyl-2-2thiazoly)-2, 5-diphenyl-2H-tetrazolium bromide (MTT) colorimetry, immunocytochemistry and transmission electron microscope, as well as western blot were employed to determine the protective effects and mechanism of Tan IIA on neuron impairment induced by Aβ_25-35_ (20µM).

## Results

TanIIA protected neurons against the neurotoxicity of Aβ_25-35,_ elevated the viability rate of neuron cells (*p* < 0.05 ), decreased the expression of p-tau in neurons induced by Aβ_25-35_, improved the cell impairment on ultramicrostructure, such as nuclear condensation and fragmentation and neurofibril collapse, keeping the normal expression of P35 on peripheral membranes, and decreased the expression of P25 in cytoplasm. TanIIA also can inhibit the translocation of Cdk5 from nuclear into cytoplasm of primary neurons and the hyperphosphorylation activity of Cdk5 induced by Aβ_25-35_.

## Conclusion

TanIIA had the protective effects on decreased cell viability rate, cytoskeletal disruption and cell loss induced by Aβ_25-35_, and this protective effect may due to that TanIIA inhibited the cleavage of P35 into P25, Cdk5 translocation from the nuclear to cytoplasm and its hyperphosphorylation activity induced by Aβ_25-35,_ and then reduced the impairment of Aβ_25-35_ on neurons through the Cdk5/P35 pathway.

